# Phase-Specific Alterations in Gut Microbiota and Their Associations with Energy Intake and Nutritional Clustering in Competitive Weightlifters

**DOI:** 10.3390/nu17203199

**Published:** 2025-10-11

**Authors:** Chun-Yu Kuo, Yu-Ching Lo, Wei-Ling Chen, Yi-Ju Hsu

**Affiliations:** 1 Graduate Institute of Sports Science, National Taiwan Sport University, Taoyuan City 33301, Taiwan; 1131301@ntsu.edu.tw (C.-Y.K.); 1141301@ntsu.edu.tw (Y.-C.L.); 2 Department of Sports Training Science-Athletics, National Taiwan Sport University, Taoyuan City 33301, Taiwan; p01@ntsu.edu.tw

**Keywords:** dietary periodization, energy intake, gut microbiota, microbial diversity, weight-class athletes

## Abstract

**Background/Objectives**: This study investigated how phase-specific dietary strategies and weight regulation influence gut microbiota composition and diversity in competitive weightlifters. Particular emphasis was placed on integrating energy intake, macronutrient clustering, and weight fluctuations across distinct training phases. **Methods**: Thirteen competitive weightlifters were recruited, with 10–12 contributing complete data per phase. Fecal and dietary samples were collected during the preparation, competition, and transition phases. Gut microbiota was profiled via 16S rRNA gene sequencing, and alpha/beta diversity was analyzed using QIIME2. K-means clustering based on caloric/macronutrient intake identified dietary patterns. Taxonomic differences were assessed using DESeq2, and microbial structures were compared across training phases, weight classes, and weight-change categories. **Results**: Overall phylum- and genus-level profiles and diversity indices remained stable across training phases, indicating community-level resilience. However, specific genera varied with dietary and physiological factors. *Enterococcus* was higher during the preparation phase, whereas *Lactobacillus* was enriched during the competition and transition phases as well as in the high-calorie cluster. Lightweight and heavyweight athletes also showed distinct microbial structures, and pre- and post-competition weight changes were associated with shifts in selected taxa. Notably, the low-calorie group exhibited higher Shannon diversity than the high-calorie group (*p* = 0.0058), with *Lactobacillus* dominance contributing to reduced evenness in high-energy diets. **Conclusions**: Despite overall microbial stability, dietary energy availability and body-weight regulation modulated specific taxa relevant to performance and recovery. By integrating dietary clustering, weight-class comparison, and pre- and post-competition weight changes, this study provides novel insight into the microbiota of resistance-trained athletes, a population underrepresented in previous research. Despite the modest sample size and single-season scope, this study offers new evidence linking dietary strategies, weight regulation, and gut microbiota in weightlifters, and highlights the need for validation in broader cohorts.

## 1. Introduction

The gut microbiota constitutes a diverse and dynamic ecosystem that plays a pivotal role in regulating metabolic processes, immune function, and overall host homeostasis [1,2]. Recent evidence suggests that gut microbes not only modulate nutrient absorption and immune responses but may also contribute to athletic performance through the production of short-chain fatty acids (SCFAs) and the maintenance of intestinal barrier integrity [3]. These mechanisms are proposed to support skeletal muscle energy utilization, reduce exercise-induced inflammation, and improve neuromuscular function, thereby facilitating endurance and recovery [4]. Moreover, exercise-induced alterations in microbial diversity and composition have been observed in both endurance [5] and strength- or team-based sports [6], reinforcing the bidirectional interaction between physical activity and the gut microbiome. Direct evidence further supports this link. For example, *Veillonella atypica* isolated from marathon runners metabolizes exercise-derived lactate into propionate, thereby enhancing performance in murine models [7]. For weightlifters, maintaining microbial stability may be particularly relevant, as gut microbes have been implicated in protein metabolism, muscle hypertrophy, and immune resilience, which are key factors influencing recovery and performance under high-intensity training demands. Taken together, such evidence highlights microbial modulation as a central mechanism mediating the physiological benefits of exercise and suggests that athlete populations provide a particularly relevant context for microbiome research.

Gut microbiota composition is shaped by multiple factors, including genetics, environment, physical activity, and especially diet, which is both modifiable and influential [8,9]. Among athletes, competitive weightlifters commonly adopt phase-specific nutritional strategies, with considerable fluctuations in energy intake and macronutrient distribution across training cycles [10]. A typical periodized model includes general preparation, competition preparation, competition, and transition phases, each imposing distinct physiological and dietary demands [11]. Energy intake is typically elevated during high-volume training in the preparation phase [12]. In contrast, the competition phase is often characterized by acute caloric restriction and weight-cutting to meet specific body mass categories [13,14]. Unlike wrestlers, boxers, or martial artists, whose weight-class demands are coupled with endurance or combat performance, weightlifters must maintain maximal strength and technical precision while undergoing rapid body-weight manipulation. This dual requirement distinguishes weightlifting as a unique model for examining how repeated cycles of caloric surplus and restriction affect the gut microbiota. Moreover, weightlifters often begin competing at a young age and are repeatedly exposed to cycles of caloric surplus and restriction throughout their careers, potentially exerting cumulative effects on the gut microbiota that remain poorly understood. Despite growing interest in athlete microbiomes, most existing studies have focused on endurance populations, leaving resistance-trained and weight-class athletes underrepresented. Addressing this gap, recent evidence from weight-class sports has shown that acute weight-cutting practices are widespread and may impose considerable physiological demands during the competition phase, potentially leading to rapid weight reduction and disruption of gut microbial stability [15,16].

In athletic populations, particularly those engaged in high-intensity or weight-class training, the impact of dietary variation on gut microbiota remains underexplored and often overlooked. Certain dietary compositions have been shown to influence gut microbial profiles by promoting the growth of bacterial taxa involved in the metabolism of available macronutrients, such as proteins and carbohydrates [17,18]. Among these, carbohydrate-rich diets have been associated with enhanced activity of saccharolytic (fermentative) bacteria, leading to increased production of short-chain fatty acids (SCFAs), which support intestinal pH balance and help attenuate inflammatory responses [19]. While such macronutrient-driven microbial shifts may provide physiological benefits, more extreme dietary practices such as severe caloric restriction and rapid weight loss, which are particularly common in weight-class athletes, have the potential to disrupt microbial homeostasis. Evidence indicates that substantial reductions in energy intake may diminish microbial diversity, compromise gut barrier integrity, impair immune responses, and increase the risk of gastrointestinal distress [20,21]. Understanding these interactions not only advances mechanistic insight but also highlights the potential of microbiome profiling to inform individualized nutrition strategies, such as targeted macronutrient adjustments or probiotic interventions, to support recovery and performance in weight-class athletes.

While extreme dietary practices may affect gut microbial stability, it remains unclear how planned dietary changes across training phases shape microbial diversity in competitive weightlifters. Most prior microbiome studies have focused on endurance athletes, leaving resistance-trained and weight-class populations underrepresented and limiting understanding of how their unique nutritional demands influence gut microbial ecology. Integrating microbiome profiling with detailed assessments of energy intake and dietary structure across defined training cycles offers a valuable opportunity to better understand the interactions between nutrition and the gut microbiota in this population. Accordingly, this investigation focused on characterizing gut microbiota dynamics across the preparation, competition, and transition phases in relation to both energy intake and overall dietary patterns. By examining phase-related microbial shifts alongside changes in nutritional intake and body weight regulation, this study aimed to clarify how dietary variation across training cycles influences gut microbial diversity and functional potential in strength athletes. We hypothesized that competitive weightlifters would exhibit distinct, phase-dependent shifts in gut microbiota composition, particularly at the genus level, that parallel variations in dietary intake and body weight regulation, with the most pronounced disruptions occurring during competition-related caloric restriction.

## 2. Materials and Methods

### 2.1. Participants

A total of 13 healthy weightlifting athletes were recruited for this study. Inclusion criteria required that participants had not consumed any lactic acid bacteria–related foods or probiotics during the three months preceding the study and had no history of food allergies, cardiovascular diseases, hypertension, metabolic disorders, or asthma. Participants were excluded if they had experienced neuromuscular injuries within the previous six months. All participants provided written informed consent before participation. The study protocol was reviewed and approved by the Institutional Review Board of Landseed International Hospital (IRB No. IRB-19-039-A2).

Prior to study initiation, an a priori sample size calculation was performed using G*Power (version 3.1.9.7; Heinrich-Heine-University, Düsseldorf, Germany). Based on a repeated-measures ANOVA design with three time points, assuming a medium effect size (f = 0.25), α = 0.05, and power (1 − β) = 0.80, the required sample size was estimated to be 12 participants. Accordingly, 13 competitive weightlifters were successfully recruited, slightly exceeding this requirement.

Consistent with the a priori sample size calculation, 10 athletes provided complete data during the preparation phase, while 12 completed the competition and transition phases. The missing participants differed across phases and were not the same individuals. Therefore, a total of 13 unique athletes contributed data to the study overall. Accordingly, blood, fecal, and dietary samples were collected from 10 athletes during the preparation phase and from 12 athletes during the competition and transition phases. For subgroup analyses (e.g., weight class, weight change, dietary clusters), paired data across relevant phases were required, and participants with incomplete records were excluded to ensure consistency. The exact number of athletes included in each analysis is reported in the corresponding tables and figure legends.

### 2.2. Experimental Design

**Figure 1:**
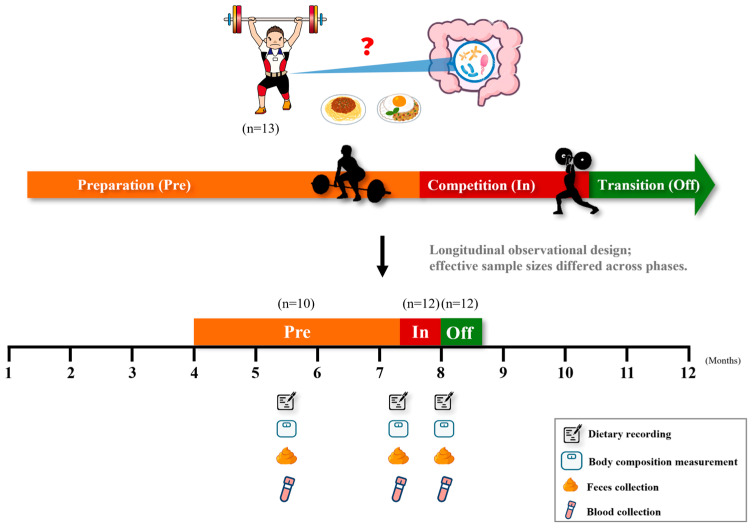
Study design and sample collection across training phases. Thirteen competitive weightlifters were enrolled and followed longitudinally across three training phases: preparation (Pre), competition (In), and transition (Off). At each phase, dietary records, body composition, fecal samples, and blood samples were collected. Effective sample sizes differed across phases due to incomplete data (Pre = 10, In = 12, Off = 12).

This study employed a longitudinal observational design (Figure 1), in which the same cohort of competitive weightlifting athletes was followed across three distinct training phases: (1) preparation (3–4 months before competition), (2) competition (from three days before to the day of competition), and (3) transition (within three days after competition). Athletes maintained their regular training routines throughout the study, consisting of six sessions per week involving both sport-specific and strength training. They were instructed to follow their habitual dietary and lifestyle practices, except for avoiding probiotic or lactic acid bacteria supplements. The use of medications and dietary supplements was documented.

As noted in Section 2.1, effective sample sizes varied slightly across phases and subgroup analyses due to scheduling conflicts and the requirement for paired data. Exact numbers are provided in the corresponding tables and figure legends.

### 2.3. Body Composition

Basic demographic information (age and sex) was collected. Body composition was assessed using a bioelectrical impedance analyzer (InBody 570, Biospace, Inc., Seoul, Republic of Korea) following an overnight fast of at least eight hours, with measurements taken during the preparation, competition, and transition phases.

### 2.4. Dietary Intake Assessment

During the preparation, competition, and transition phases, participants were instructed to complete three days of dietary records within the 3–4 days preceding each fecal sample collection, including the collection day itself. Participants photographed all foods and beverages consumed, including meals, snacks, drinks, and dietary supplements. For packaged foods, participants were asked to provide the brand name, product weight, and nutrition label. Prior to the study, participants received detailed instructions on how to complete the dietary record and were asked to maintain their habitual diet during the recording period. The three-day window was designed to capture both training and non-training days of varying intensity whenever possible, thereby reflecting typical intake under real-world training conditions. A nutritionist converted the dietary data into daily energy intake and macronutrient composition (carbohydrates, proteins, and fats) using the Taiwan Food Nutrition Database provided by the Ministry of Health and Welfare (MOHW, Taipei, Taiwan) [22], and all entries were further cross-checked by a registered dietitian to ensure accuracy. Analyses focused on daily energy and macronutrient composition, whereas micronutrients were not assessed, as the study specifically aimed to capture dietary factors most relevant to energy availability and gut microbiota.

For dietary intake and gut microbiota comparisons, analyses focused on the competition and transition phases, which represent critical periods of dietary manipulation in weight-class athletes. In total, 24 dietary records were expected (12 from the competition phase and 12 from the transition phase). However, one athlete did not provide competition-phase data, and another did not provide transition-phase data. To ensure consistency, both competition and transition records from these athletes were excluded, resulting in 22 valid records from 11 participants.

To classify dietary patterns, k-means clustering was performed using standardized (Z-score) values of daily energy intake and macronutrient distribution (carbohydrates, proteins, and fats). The optimal number of clusters was evaluated using the elbow method, which assesses the reduction in within-cluster sum of squares as cluster number increases. Based on this evaluation, three clusters (k = 3) were selected as this provided the most appropriate balance between statistical robustness and nutritional interpretability, thereby grouping dietary records into high-, moderate-, and low-calorie intake patterns. Based on total energy intake, these 22 records were classified into three dietary clusters: group 1 (G1; high-calorie, n = 10), group 2 (G2; moderate-calorie, n = 8), and group 3 (G3; low-calorie, n = 4). The average macronutrient composition and energy intake were as follows: G1, 44.6% carbohydrates, 18.5% proteins, and 37.0% fats, with 1859.2 kcal/day; G2, 44.4% carbohydrates, 18.4% proteins, and 35.1% fats, with 1231.1 kcal/day; and G3, 45.7% carbohydrates, 18.5% proteins, and 35.8% fats, with 449.7 kcal/day. Further analyses were conducted to examine associations between energy intake and gut microbiota diversity.

### 2.5. Blood Sample Collection and Analysis

Venous blood samples were obtained from each participant at every study phase after an overnight fast of at least 8 h. Whole blood was centrifuged at 3000 rpm for 15 min at 4 °C using a refrigerated centrifuge to separate serum. Serum concentrations of glucose (GLU), blood urea nitrogen (BUN), creatinine, uric acid (UA), glutamic oxaloacetic transaminase (GOT), glutamic pyruvic transaminase (GPT), creatine phosphokinase (CPK), lactate dehydrogenase (LDH), and ammonia (NH_3_) were measured using an automated biochemical analyzer (Hitachi 7070A; Hitachi High-Technologies Corp., Tokyo, Japan).

### 2.6. Faecal Specimen Collection

For each study phase, participants collected one faecal sample using a sterile collection tube prefilled with DNA/RNA Shield (Zymo Research, Irvine, CA, USA) and equipped with a spoon attached to the screw cap, into which approximately 0.5 g of stool was transferred. The tube was then tightly sealed and shaken for 30 s to homogenize the stool with the preservation solution, before being placed in a plastic biohazard bag for transport. Samples were self-collected at home and delivered to the study investigator on the same day. If defecation occurred the evening before the scheduled visit, participants were instructed to temporarily store the sample in a household refrigerator (~5 °C) and deliver it the following morning. Upon receipt, all specimens were logged and immediately stored at −80 °C. Across all collections, the interval between defecation and freezing did not exceed 24 h. All samples were handled under standardized conditions to ensure proper preservation and subsequent processing for microbial DNA extraction.

### 2.7. DNA Extraction

Fresh fecal samples were suspended in phosphate-buffered saline (PBS) at a ratio of 1:9 and vortexed until a homogeneous suspension was obtained. A 200 μL homogenized suspension was then washed twice with 1 mL of PBS and centrifuged at 16,200× *g* for 5 min, and 1 mL of the supernatant was discarded. Fecal DNA was subsequently extracted using a modified method developed by Zhu Heng et al. [23]. To the 200 μL washed fecal suspension, 700 mg of glass beads (product No. 11079101, 0.1 mm; BioSpec Products, Bartlesville, OK, USA), 250 μL of extraction buffer (containing 100 mM Tris-HCl, 40 mM EDTA, pH 9.0), and 500 μL of the phenol-chloroform isoamyl alcohol mixture (product No. 77617, Sigma-Aldrich, St. Louis, MO, USA) were added. The mixture was then homogenized at 6.5 m/s for 30 s using FastPrep-24™ (MP Biomedicals, Irvine, CA, USA). After homogenization, the mixture was added to 50 μL of 10% SDS and heated at 50 °C for 20 min. Next, it was mixed with 150 μL of 3 M sodium acetate on ice for 5 min and centrifuged at 16,200× *g* for 5 min at 4 °C. The supernatant was transferred to a new 1.5 mL centrifuge tube and mixed with 450 μL of isopropanol to precipitate DNA, followed by centrifugation at 16,200× g for 10 min at 4 °C. The DNA pellet was washed twice in 70% ethanol and centrifuged at 16,200× *g* for 1 min at 4 °C. After centrifugation, the supernatant was discarded, and the DNA pellet was heated at 65 °C for 10 to 15 min. The dried DNA pellet was then resuspended in 30 μL of ddH_2_O and stored at −20 °C until further analysis.

### 2.8. 16S rRNA Gene Sequencing and Bioinformatics Processing

The V3-V4 region of the 16S rRNA gene was amplified using specific primers (341F: 5′-CCTACGGGNGGCWGCAG-3′ and 805R: 5′-GACTACHVGGGTATCTAATCC-3′) following the 16S Metagenomic Sequencing Library Preparation procedure (Illumina, San Diego, CA, USA). Amplicon pools were sequenced on the Illumina MiSeq™ sequencing platform (Illumina, San Diego, CA, USA). Bioinformatic analysis was conducted with QIIME2 (version 2021.2; https://qiime2.org, accessed on 15 August 2025) as previously described [24]. Based on the characteristics of the compositional data, networks of specific families in each group were built using SparCC (https://bitbucket.org/yonatanf/sparcc, accessed on 15 August 2025) correlation coefficients [25]. The networks were visualized using Cytoscape (version 3.8.2; https://github.com/cytoscape/cytoscape/releases/3.8.2/, accessed on 23 November 2021). Correlations between the relative abundances of taxa and exercise performance indices and total amino acids were performed using Spearman correlation. The Kyoto Encyclopedia of Genes and Genomes (KEGG; https://www.genome.jp/kegg/, accessed on 6 December 2021) database was used to analyze pathway enrichment, utilizing Phylogenetic Investigation of Communities by Reconstruction of Unobserved States (PICRUSt2; version 2.5.0; https://github.com/picrust/picrust2, accessed on 15 August 2025). The raw sequence files supporting the findings of this article are deposited in the NCBI Sequence Read Archive (SRA) database, with the project accession number PRJNA953842.

### 2.9. Statistical Analysis

All data are presented as mean ± standard deviation (SD). One-way analysis of variance (ANOVA) was employed to compare body composition, macronutrient and energy intake, as well as blood biochemical parameters across different training phases, using SPSS Statistics for Windows, Version 20.0 (IBM Corp., Armonk, NY, USA). For gut microbiota analyses, non-parametric tests were used due to the non-normal distribution of microbial data. Alpha diversity metrics, including Observed Species and the Shannon index, were calculated using QIIME2 (version 2021.2; https://qiime2.org, accessed on 15 August 2025) and compared across training phases, weight classes, weight-change groups, and dietary intake clusters using the Kruskal–Wallis test. Pairwise comparisons were conducted using the Wilcoxon rank-sum test. Beta diversity was assessed based on both weighted and unweighted UniFrac distances and visualized using Principal Coordinates Analysis (PCoA). Group-level differences in microbial community structure across training phases, weight classes, weight-change groups, and dietary intake clusters were evaluated using permutational multivariate analysis of variance (PERMANOVA). To identify differentially abundant taxa, the DESeq2 package (version 1.38.0) in R software (version 4.3.2; R Foundation for Statistical Computing, Vienna, Austria) was used. This method models microbial count data with a negative binomial distribution while adjusting for sample variance. Genera present in less than 25% of samples were excluded prior to analysis to reduce zero-variance errors and spurious significance. This prevalence filtering step was applied to minimize the impact of zero-inflated features, which are common in microbiome datasets. Comparisons were conducted across training phases (preparation, competition, transition), weight-change groups (lightweight ≤ 64 kg vs. heavyweight ≥ 67 kg), dietary intake clusters (high-calorie vs. low-calorie), and pre- and post-competition weight-change groups (gain vs. loss). Wald tests were used to evaluate differential abundance, and genera with a Benjamini–Hochberg adjusted *p*-value < 0.05 (false discovery rate [FDR] < 5%) were considered statistically significant. K-means clustering analysis was performed in R software (version 4.3.2; R Foundation for Statistical Computing, Vienna, Austria) to classify dietary intake patterns based on total energy and macronutrient distribution. Correlations between microbial taxa and macronutrient intake were evaluated using Spearman’s rank correlation. All statistical tests were two-tailed, and a significance level of *p* < 0.05 was applied throughout the study. For subgroup analyses, athletes were categorized into two groups (lightweight ≤ 64 kg; heavyweight ≥ 67 kg) to reflect practical distinctions in weight-class sports, and into weight gain vs. weight loss groups to capture the direction of short-term weight manipulation before and after competition. Because paired data were required, participants with incomplete measurements in the relevant phases were excluded, leading to slight variations in sample size across analyses. The exact number of athletes included in each comparison is reported in the corresponding tables and figure legends. These variations in n reflect differences in data completeness across phases rather than additional recruitment.

## 3. Results

### 3.1. Characteristics of the Study Population

Table 1 presents the characteristics of the study participants. A total of 13 unique athletes were recruited, but complete data were not available for all phases. Specifically, 10 athletes provided data during the preparation phase, while 12 athletes contributed to the competition and transition phases. The missing participants differed across phases and were not the same individuals. No significant differences were observed in body composition variables, including body weight, skeletal muscle mass, and fat mass, across the three training phases (*p* > 0.05).

### 3.2. Macronutrient and Energy Intake Across Training Phases

Data were analyzed according to the available samples in each phase. Table 2 summarizes the average dietary intake across three-day periods during each training phase. Significant differences were observed in daily total energy intake per kilogram of body weight across the three phases (F (2, 29) = 4.963, *p* = 0.014, η^2^ = 0.255). Post-hoc Tukey HSD tests revealed that energy intake was significantly higher during the transition phase compared to the competition phase (*p* = 0.012). Protein intake (F (2, 29) = 3.942, *p* = 0.031, η^2^ = 0.214) and fat intake (F (2, 29) = 5.562, *p* = 0.009, η^2^ = 0.277) also differed significantly across phases, with both macronutrients being significantly higher in the transition phase than in the competition phase (*p* = 0.042 and *p* = 0.006, respectively). Carbohydrate intake was higher in the transition phase than in the competition phase, but this difference was not statistically significant (*p* = 0.156). The percentage of energy intake from protein (*p* = 0.195) and fat (*p* = 0.055) tended to be higher during the transition phase compared to the competition phase, although the differences did not reach statistical significance. Conversely, the percentage of energy intake from carbohydrates tended to be lower during the transition phase, but this difference was also not statistically significant (*p* = 0.261).

### 3.3. Blood Biochemical Parameters Across Training Phases

Data were analyzed according to the available samples in each phase. Table 3 summarizes the changes in blood biochemical parameters across the three training phases. Significant differences were observed in glucose levels across the three phases (F (2, 31) = 4.596, *p* = 0.018, η^2^ = 0.229). Post-hoc Tukey HSD tests revealed that glucose concentrations were significantly higher during the preparation phase compared to the competition phase (*p* = 0.019). Significant differences were observed in lactate levels across the three phases (F (2, 31) = 43.184, *p* < 0.001, η^2^ = 0.736). Post-hoc Tukey HSD tests revealed that lactate concentrations were significantly lower during the transition phase compared to the competition phase (*p* = 0.009), and significantly lower during the competition phase compared to the preparation phase (*p* < 0.001), indicating a progressive decrease across phases (off < in < pre). Similarly, significant differences were also observed in LDH levels across the three phases (F (2, 31) = 21.870, *p* < 0.001, η^2^ = 0.585). Post-hoc Tukey HSD tests indicated that LDH concentrations were significantly lower during the transition phase compared to the competition phase (*p* = 0.001), and significantly lower during the competition phase compared to the preparation phase (*p* = 0.027), suggesting a consistent decreasing trend (off < in < pre).

Ammonia (NH_3_) levels also differed significantly across phases (F (2, 31) = 12.990, *p* < 0.001, η^2^ = 0.456). Concentrations were significantly lower during the competition phase compared to both the preparation (*p* < 0.001) and transition phases (*p* = 0.008), while no difference was found between the preparation and transition phases (*p* = 0.150), suggesting a transient reduction during competition. No statistically significant differences were detected in BUN, creatinine, UA, GOT, GPT, or CPK levels across the phases (*p* > 0.05 for all).

### 3.4. Gut Microbiota Composition Across Training Phases

**Figure 2:**
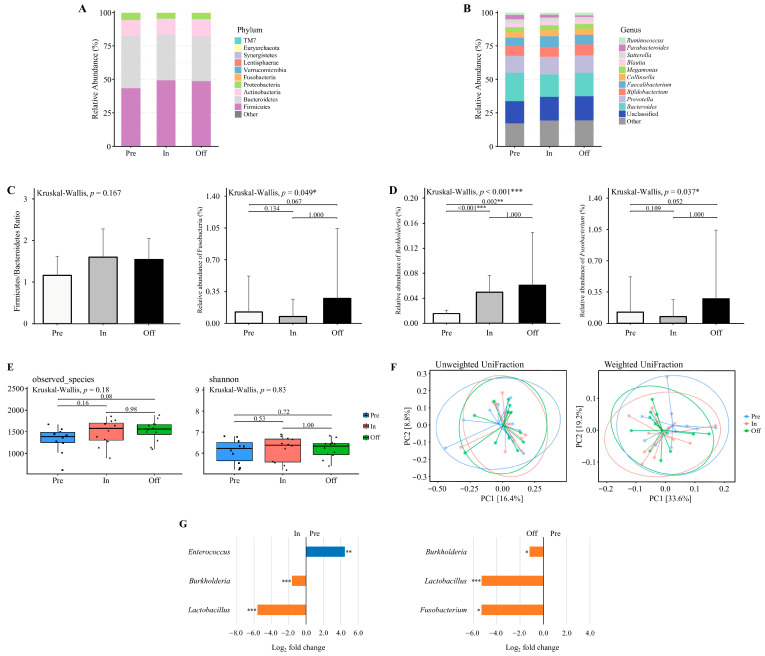
The diversity and composition of the gut microbiome across training phases. (**A**,**B**) The relative abundance of major bacterial taxa at the (**A**) phylum and (**B**) genus levels across three training phases. (**C**) Firmicutes to Bacteroidetes (F/B) ratio and the relative abundance of *Fusobacteria* at the phylum level. Only *Fusobacteria* exhibited a significant difference across phases. (**D**) Relative abundance of genera with significant phase-specific differences, including *Burkholderia* and *Fusobacterium*. (**E**) Alpha diversity assessed by observed species and Shannon index. (**F**) Beta diversity visualized using principal coordinate analysis (PCoA) based on unweighted UniFrac and weighted UniFrac distances. Explained variance of PC1 and PC2 is indicated as percentages on each axis. * *p* < 0.05, ** *p* < 0.01, *** *p* < 0.001. (**G**) Differential abundance of bacterial taxa between Pre vs. In and Pre vs. Off phases. Taxa with Benjamini–Hochberg corrected *p*-value below 0.05 are shown. * *padj* < 0.05, ** *padj* < 0.01, *** *padj* < 0.001. Preparation (pre, n = 10), Competition (in, n = 12), Transition (off, n = 12).

Data were analyzed according to the available samples in each phase. Figure 2A and Figure 2B illustrate the relative abundance of gut microbiota across different training phases at the phylum and genus levels, respectively. Distinct shifts in microbial composition were observed at both taxonomic levels. At the phylum level, the dominant phyla included *Firmicutes*, *Bacteroidetes*, *Actinobacteria*, *Proteobacteria*, *Fusobacteria,* and *Verrucomicrobia* (Figure 2A). The Firmicutes to Bacteroidetes (F/B) ratio did not differ significantly across the training phases (Kruskal–Wallis, *p* = 0.167; Figure 2C). Although the relative abundance of *Fusobacteria* was significantly different among the phases (Kruskal–Wallis, *p* = 0.049; Figure 2C), post hoc Dunn’s test did not identify any significant pairwise differences (*p* > 0.05).

At the genus level, the dominant genera were *Bacteroides*, *Prevotella*, *Bifidobacterium*, *Faecalibacterium*, *Collinsella*, and *Megamonas* (Figure 2B). The relative abundance of *Burkholderia* differed significantly among the phases (Kruskal–Wallis, *p* < 0.001; Figure 2D), with significantly higher levels observed in the competition and transition phases compared to the preparatory phase (*p* = 0.002 and *p* < 0.001, respectively). Similarly, *Fusobacterium* abundance varied significantly across phases (Kruskal–Wallis, *p* = 0.037; Figure 2D), though Dunn’s post hoc test did not detect any significant pairwise differences (*p* > 0.05).

### 3.5. Alpha and Beta Diversity Analysis Across Training Phases

Alpha diversity indices (Observed Species and Shannon index) were assessed across training phases using microbiota data from 10 athletes in the preparation phase, 12 athletes in the competition phase, and 12 athletes in the transition phase (Figure 2E). No significant differences were detected in microbial richness and diversity between phases (*p* > 0.05). Beta diversity was examined using principal coordinates analysis (PCoA) based on weighted and unweighted UniFrac distances (Figure 2F). No significant clustering was observed among training phases (*p* > 0.05), indicating that gut microbial community structures remained stable across training periods.

### 3.6. Taxonomic Abundance Changes Across Training Phases

Differential abundance analysis was conducted on microbiota data from 10 athletes in the preparation phase, 12 athletes in the competition phase, and 12 athletes in the transition phase (Figure 2G). Results revealed a significantly higher relative abundance of Enterococcus in the preparation phase compared to the competition phase, whereas *Burkholderia* and *Lactobacillus* were significantly lower (adjusted *p* < 0.05). No genera demonstrated statistically significant differences between the competition and transition phases, while comparisons between the preparation and transition phases revealed significantly lower abundances of *Burkholderia*, *Lactobacillus*, and *Fusobacterium* in the preparation phase (adjusted *p* < 0.05). Although several other genera displayed large log_2_ fold changes (>2.0 or <−2.0), these did not reach statistical significance and are reported in Tables S1–S3. These shifts indicate that *Enterococcus* may be particularly responsive to dietary protein conditions, while the enrichment of *Lactobacillus* during the competition and transition phases suggests a potential role in carbohydrate-associated adaptation and recovery processes.

### 3.7. Alpha and Beta Diversity Analysis Across Weight Classes

**Figure 3:**
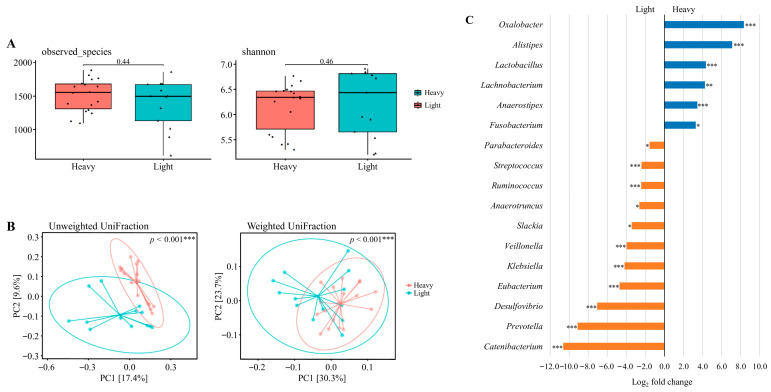
The diversity and composition of the gut microbiome across weight classes. (**A**) Alpha diversity assessed by observed species and Shannon index. (**B**) Beta diversity visualized using principal coordinate analysis (PCoA) based on unweighted UniFrac and weighted UniFrac distances. Explained variance of PC1 and PC2 is indicated as percentages on each axis. * *p* < 0.05, ** *p* < 0.01, *** *p* < 0.001. (**C**) Differential abundance of bacterial taxa between heavy and light weight classes. Taxa with Benjamini–Hochberg corrected *p*-value below 0.05 are shown. * *padj* < 0.05, ** *padj* < 0.01, *** *padj* < 0.001. Data were derived from the preparation, competition, and transition phases. A total of 34 datasets were originally available. After excluding one athlete who did not provide competition-phase samples (and the corresponding preparation and transition data), 31 valid datasets remained: lightweight (n = 18) and heavyweight (n = 13).

Data derived from 31 valid datasets after excluding one athlete who did not provide competition-phase samples. As shown in Figure 3A, alpha diversity was assessed using Observed Species and the Shannon index. No statistically significant differences were observed in microbial richness or diversity between the light and heavy groups (Wilcoxon rank-sum test; *p* = 0.44 and *p* = 0.46, respectively).

Beta diversity was further evaluated using principal coordinates analysis (PCoA) based on unweighted and weighted UniFrac distances (Figure 3B). Clear clustering patterns were observed between the groups, with significant differences in community composition detected for both unweighted (*p* < 0.001) and weighted (*p* < 0.001) UniFrac distances, indicating distinct microbial structures between lightweight and heavyweight athletes.

### 3.8. Taxonomic Abundance Changes Across Weight Classes

Differential abundance analysis was performed on the 31 valid datasets (lightweight n = 18, heavyweight n = 13) derived from the preparation, competition, and transition phases (Figure 3C). The results revealed a significantly higher relative abundance of *Oxalobacter*, *Alistipes*, *Lactobacillus*, *Lachnobacterium*, *Anaerostipes*, and *Fusobacterium* in the heavy weight group compared to the light weight group, whereas *Parabacteroides*, *Streptococcus*, *Ruminococcus*, *Anaerotruncus*, *Slackia*, *Veillonella*, *Klebsiella*, *Eubacterium*, *Desulfovibrio*, *Prevotella*, and *Catenibacterium* were significantly lower (adjusted *p* < 0.05). Several additional genera exhibited large log_2_ fold changes (>2.0 or <−2.0), but these differences were not statistically significant and are summarized in Table S4. These class-specific patterns may reflect long-term differences in dietary energy intake and body composition demands across weight categories.

### 3.9. Alpha and Beta Diversity Analysis Across to Pre- and Post-Competition Weight Change

**Figure 4:**
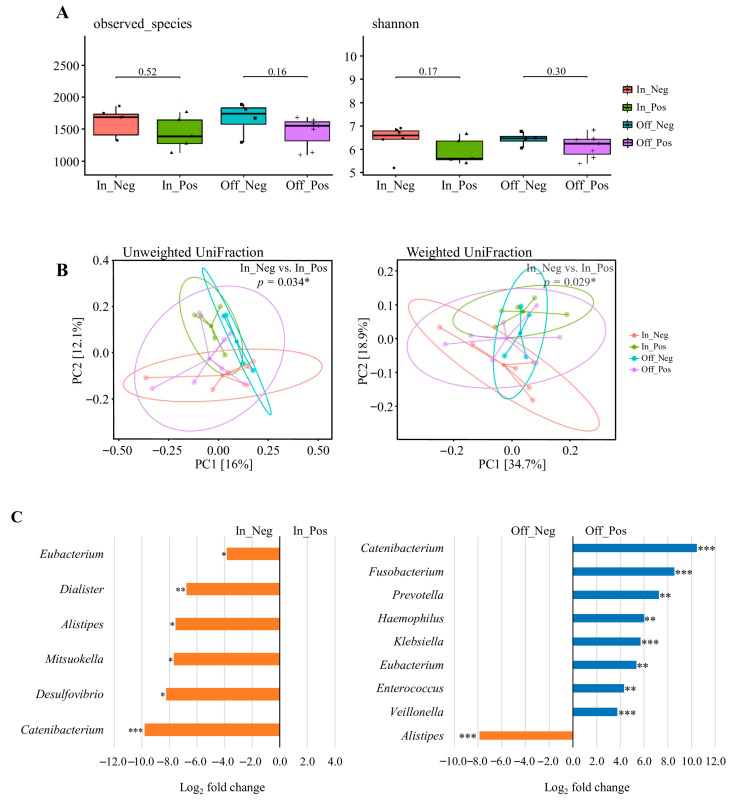
The diversity and composition of the gut microbiome across weight change. (**A**) Alpha diversity assessed by observed species and Shannon index. (**B**) Beta diversity visualized using principal coordinate analysis (PCoA) based on unweighted UniFrac and weighted UniFrac distances. Explained variance of PC1 and PC2 is indicated as percentages on each axis. * *p* < 0.05, ** *p* < 0.01, *** *p* < 0.001. (**C**) Differential abundance of bacterial taxa between In_Pos vs. In_Neg and Off_Pos vs. Off_Neg weight change. Taxa with Benjamini–Hochberg corrected *p*-value below 0.05 are shown. * *padj* < 0.05, ** *padj* < 0.01, *** *padj* < 0.001. Data were derived from the competition (n = 11) and transition (n = 11) phases, resulting in 22 datasets. Participants were categorized into four groups according to the direction of body-weight change: In_Pos (n = 5), In_Neg (n = 6), Off_Pos (n = 7), and Off_Neg (n = 4). “In” groups were defined by comparing competition weight with official weigh-in body weight, whereas “Off” groups were defined by comparing official weigh-in body weight with transition-phase body weight. One athlete did not provide competition-phase data; therefore, this individual’s transition data were also excluded to ensure consistency, resulting in the final sample sizes shown above.

Data were derived from the competition (n = 11) and transition (n = 11) phases, resulting in 22 datasets, which were stratified into four weight-change categories: In_Pos (n = 5), In_Neg (n = 6), Off_Pos (n = 7), and Off_Neg (n = 4) (Figure 4C). As shown in Figure 4A, alpha diversity indices (Observed Species and Shannon index) were compared between the paired subgroups. No significant differences were observed in microbial richness or diversity between the paired subgroups (Wilcoxon rank-sum test, all *p* > 0.05).

Beta diversity was assessed using principal coordinates analysis (PCoA) based on unweighted and weighted UniFrac distances (Figure 4B). Significant separation between In_Pos and In_Neg for both unweighted (*p* = 0.034) and weighted (*p* = 0.029) UniFrac metrics, indicating that the direction of pre-competition weight change was associated with compositional shifts in the gut microbiota. By contrast, no significant differences were observed between Off_Pos and Off_Neg (all *p* > 0.05), suggesting that post-competition weight fluctuations had a lesser impact on overall community structure.

### 3.10. Taxonomic Abundance Changes Across to Pre- and Post-Competition Weight Change

Differential abundance analysis was performed using the 22 valid datasets derived from the competition (n = 11) and transition (n = 11) phases, which were stratified into four weight-change categories: In_Pos (n = 5), In_Neg (n = 6), Off_Pos (n = 7), and Off_Neg (n = 4) (Figure 4C). The results revealed a significantly lower relative abundance of *Eubacterium*, *Dialister*, *Alistipes*, *Mitsuokella*, *Desulfovibrio*, and *Catenibacterium* in the In_Pos group compared to the In_Neg group (adjusted *p* < 0.05). When comparing the Off_Pos group and Off_Neg group, the relative abundances of *Catenibacterium*, *Fusobacterium*, *Prevotella*, *Haemophilus*, *Klebsiella*, *Eubacterium*, *Enterococcus*, and *Veillonella* were significantly higher in the Off_Pos group, whereas *Alistipes* was significantly lower in the Off_Pos group (adjusted *p* < 0.05). Additional genera showing large log_2_ fold changes (>2.0 or <−2.0), but these differences were not statistically significant and are summarized in Tables S5 and S6. These results suggest that acute pre- and post-competition weight changes drive taxon-specific alterations in the gut microbiota, highlighting their potential role as markers of nutritional adaptation and physiological stress in weight-class athletes.

### 3.11. Impact of Dietary Intake on Gut Microbiota Diversity

Gut microbiota data were analyzed in relation to dietary intake clusters derived from paired competition and transition phases. Originally, 24 dietary datasets (12 competition and 12 transition) were expected. However, one athlete did not provide competition-phase data, and another did not provide transition-phase data. To ensure consistency, both competition and transition records from these athletes were excluded, leaving 22 valid datasets from 11 athletes for cluster analysis.

**Figure 5:**
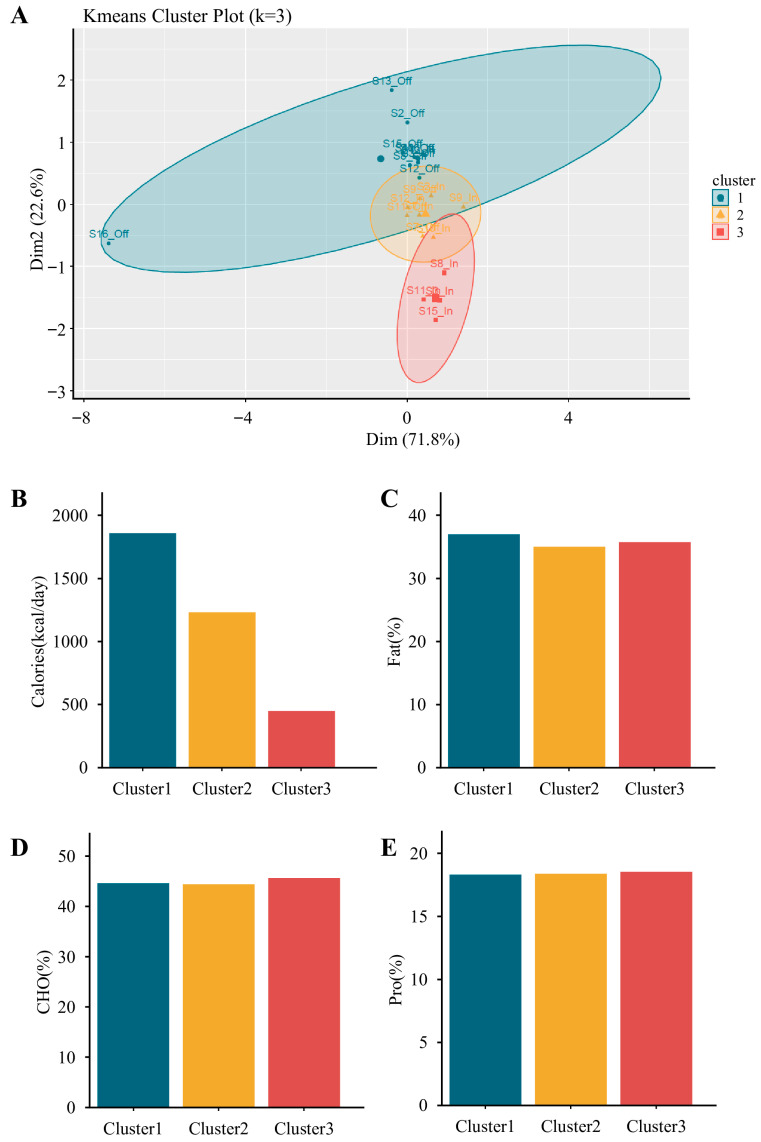
K-means clustering of participants based on energy and macronutrient intake profiles. (**A**) K-means cluster plot (k = 3) visualizing three distinct dietary clusters from paired competition and transition phases (originally n = 24, reduced to n = 22 after excluding one athlete missing competition data and one missing transition data). (**B**–**E**) Comparisons of dietary intake characteristics among clusters, including (**B**) total energy intake (kcal/day), (**C**) fat (% of total energy), (**D**) carbohydrate (% of total energy), and (**E**) protein (% of total energy). Cluster 1 (n = 10) was characterized by the highest caloric intake, Cluster 2 (n = 8) showed moderate intake, and Cluster 3 (n = 4) showed the lowest. Macronutrient distribution appeared similar across clusters, except for total energy.

Participants were classified into three dietary intake clusters using k-means analysis: Cluster 1 (high-calorie: 44.6% carbohydrates, 18.3% protein, 37% fat; total energy: 1859.2 kcal), Cluster 2 (moderate-calorie: 44.4% carbohydrates, 18.4% protein, 35.1% fat; total energy: 1231.1 kcal), and Cluster 3 (low-calorie: 45.7% carbohydrates, 18.6% protein, 35.8% fat; total energy: 449.7 kcal) (Figure 5). In the competition phase, 2 participants clustered into the high-calorie group (G1), 5 into the moderate-calorie group (G2), and 4 into the low-calorie group (G3). In the transition phase, 8 participants clustered into G1, 3 into G2, and none into G3. These distributions clarify the subgroup sizes used in subsequent analyses.

**Figure 6:**
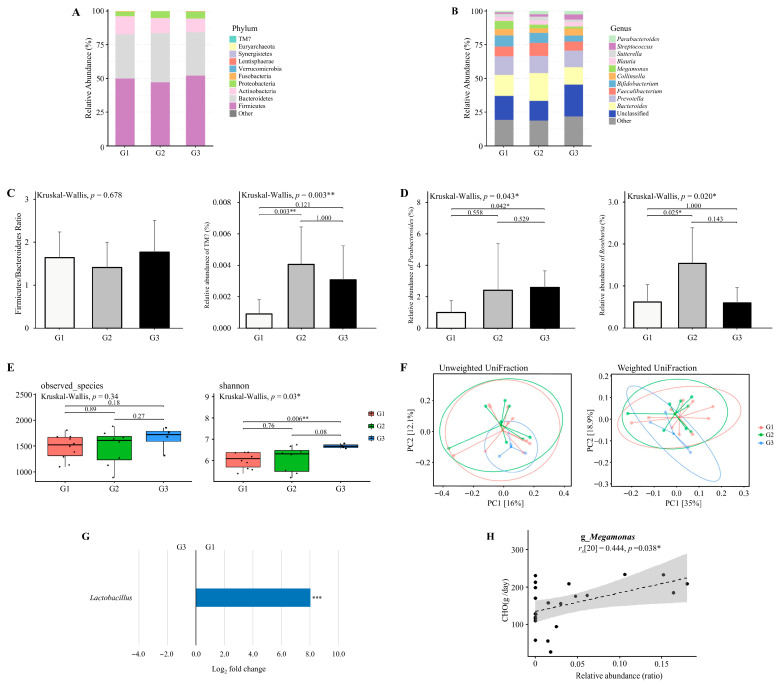
The diversity and composition of the gut microbiome across dietary intake clusters. (**A**,**B**) The relative abundance of major bacterial taxa at the (**A**) phylum and (**B**) genus levels across three dietary intake clusters derived from paired competition and transition phases. Originally 24 datasets were expected (12 competition and 12 transition), but one athlete did not provide competition data and another did not provide transition data. To maintain paired analyses, both phases from these athletes were excluded, leaving 22 valid datasets (n = 22; Cluster 1 = 10, Cluster 2 = 8, Cluster 3 = 4). (**C**) Firmicutes to Bacteroidetes (F/B) ratio and the relative abundance of *TM7* at the phylum level. Only *TM7* exhibited a significant difference across clusters. (**D**) Relative abundance of genera with significant group-specific differences, including *Parabacteroides* and *Roseburia*. (**E**) Alpha diversity assessed by observed species and Shannon index. (**F**) Beta diversity visualized using principal coordinate analysis (PCoA) based on unweighted UniFrac and weighted UniFrac distances. Explained variance of PC1 and PC2 is indicated as percentages on each axis. * *p* < 0.05, ** *p* < 0.01, *** *p* < 0.001. (**G**) Differential abundance of bacterial taxa between G1 and G3 group. Taxa with Benjamini–Hochberg corrected *p*-value below 0.05 are shown. (**H**) Association between the relative abundance of *Megamonas* and carbohydrate intake (g/day). * *padj* < 0.05, ** *padj* < 0.01, *** *padj* < 0.001.

Figure 6A,B show the relative abundance of gut microbiota across clusters. The dominant phyla and genera were consistent with those identified across training phases (Section 3.4), with *Firmicutes*, *Bacteroidetes*, *Actinobacteria*, *Proteobacteria*, *Fusobacteria*, *Verrucomicrobia*, and genera such as *Bacteroides*, *Prevotella*, *Faecalibacterium*, *Bifidobacterium*, *Collinsella*, and *Megamonas* prevailing across clusters. (Figure 6A,B).

The Firmicutes to Bacteroidetes (F/B) ratio did not differ significantly across dietary intake clusters (Kruskal–Wallis, *p* = 0.678; Figure 6C). The relative abundance of *TM7* differed significantly among the clusters (Kruskal–Wallis, *p* = 0.003; Figure 6C), with significantly higher levels observed in the moderate-calorie group (G2) compared to the high-calorie group (G1) (*p* = 0.003).

The relative abundance of *Parabacteroides* differed significantly among the clusters (Kruskal–Wallis, *p* = 0.043; Figure 6D), with significantly higher levels observed in the low-calorie group (G3) compared to the high-calorie group (G1) (*p* = 0.042). The relative abundance of *Roseburia* differed significantly among the clusters (Kruskal–Wallis, *p* = 0.020; Figure 6D), with significantly higher levels observed in the moderate-calorie group (G2) compared to the high-calorie group (G1) (*p* = 0.025).

Alpha diversity analysis revealed a significantly higher Shannon index in the low-calorie group (G3) compared to the high-calorie group (G1) (*p* = 0.006; Figure 6E). However, beta diversity analysis based on weighted and unweighted UniFraction distances showed no significant clustering (*p* > 0.05; Figure 6F).

### 3.12. Differential Abundance Analysis Between High- and Low-Calorie Groups

Differential abundance analysis was conducted using the paired datasets retained for dietary intake clusters (n = 22; G1 = 10, G2 = 8, G3 = 4). As shown in Figure 6G, there is a significantly higher relative abundance of *Lactobacillus* in the high-calorie group (G1) compared to the low-calorie group (G3) (adjusted *p* < 0.05). Several additional genera exhibited large log_2_ fold changes (>2.0 or <−2.0), but these differences were not statistically significant and are summarized in Table S7. The enrichment of *Lactobacillus* in the high-calorie group indicates a potential link between energy surplus and the proliferation of carbohydrate-fermenting taxa.

### 3.13. Association Between Specific Bacterial Genera and Macronutrient or Energy Intake

Associations between bacterial genera and dietary intake variables were assessed using Spearman’s correlation based on the same 22 paired datasets. As illustrated in Figure 6H, *Megamonas* showed a positive correlation with carbohydrate intake (*r_s_* [20] = 0.444, *p* = 0.038). No other genera demonstrated statistically significant correlations with macronutrient or energy intake.

## 4. Discussion

This study examined the interplay between training-phase-specific dietary strategies, body weight regulation, and gut microbiota composition in competitive weightlifters. Although significant variations in energy and macronutrient intake were observed across phases, the gut microbiota showed overall stability at the broader community scale, with no significant differences detected in phylum- or genus-level profiles, alpha diversity, or beta diversity indices. Nevertheless, differential abundance analyses revealed phase-associated shifts in certain bacterial genera, and community composition varied by weight class and body-weight fluctuation. Taken together, these results suggest that while the gut microbiota in weightlifters may demonstrate resilience at the global community level, it also undergoes targeted adjustments associated with physiological status and energy availability. This pattern is consistent with athlete studies showing stable community profiles but sport- or diet-related shifts in specific taxa, such as increased *Prevotella* in cyclists consuming carbohydrate-rich diets [26] and *Veillonella* enrichment in endurance runners following exercise [7]. Comparable observations have also been reported in rugby players, where overall diversity remained stable while diet and training status shaped the abundance of particular genera [27]. Notably, recent studies of weightlifters have identified distinctive gut microbial and metabolic profiles that separate strength athletes from endurance counterparts, highlighting compositional signatures aligned with anaerobic versus aerobic energy system demands [28,29]. Such evidence underscores that the microbiota of strength- and weight-class athletes is not randomly variable but reflects the physiological and nutritional pressures of their sport. In line with these reports, our findings suggest that although community-level stability was preserved, weightlifters exhibited specific microbial adjustments corresponding to dietary restriction during competition and increased energy availability during transition. This pattern may reflect ecological resilience, where functional redundancy ensures that overall community diversity is maintained, even as specific taxa shift in response to dietary and physiological pressures.

Consistent with nutritional periodization practices in weight-class sports, dietary energy and protein intake were significantly lower during the competition phase compared to the transition phase, consistent with pre-weigh-in weight-control strategies [13,14]. The post-competition transition phase featured higher energy and protein intake, in line with guidelines that support muscle recovery and metabolic restoration [12]. Experimental work has shown that protein consumption stimulates muscle protein synthesis, contributing to training-related accrual of lean mass over time [30,31], and meta-analyses have indicated that resistance training combined with protein supplementation is associated with greater gains in muscular strength and hypertrophy [32,33]. Overall, these observations are consistent with evidence that increased energy and protein availability after competition facilitates recovery and muscle remodeling.

Importantly, these intake patterns coincided with microbial changes, as *Enterococcus* was higher in the preparation phase than in the competition phase. This observation is consistent with reports that high-protein diets can favor *Enterococcus* proliferation [34,35,36], suggesting that *Enterococcus* may be particularly responsive to dietary protein manipulation in weight-class athletes. Together, the dietary and microbial findings highlight the importance of considering protein-focused strategies not only for muscle adaptation but also for their ecological impact on the gut microbiota. Beyond protein-related shifts, carbohydrate availability may also shape microbial dynamics. *Lactobacillus* was more abundant during the competition and transition phases than in the preparation phase. This pattern may reflect dietary conditions favoring its proliferation, such as a higher proportion of carbohydrate intake during competition and a relative relaxation of dietary restrictions during transition. Previous studies have described *Lactobacillus* as a versatile fermenter that supports gut health [19,37,38], and its consistent enrichment across phases suggests it could represent a characteristic genus within the microbiota of weight-class athletes.

Blood biochemical results revealed patterns that paralleled the observed dietary differences. BUN showed a non-significant upward trend in the competition phase compared with preparation (*p* = 0.064), which may reflect transient changes in protein metabolism or hydration status. CK levels also did not change significantly, but their marked interindividual variability suggests heterogeneous responses in muscle stress and recovery status among athletes, a phenomenon frequently reported in strength and weight-class sports [39]. These findings are consistent with the nutritional strategies employed, where lower energy and protein intake during competition may increase reliance on endogenous substrates, while higher intake during transition supports recovery. In contrast, lactate and LDH concentrations declined progressively from preparation to competition to transition (all *p* < 0.001), indicating reduced acute metabolic strain across phases and aligning with the shift toward nutritional and physiological restoration during the post-competition period.

In addition to training-phase effects, we observed important differences in gut microbiota by weight class. Although alpha diversity did not differ significantly, beta diversity showed clear clustering between lightweight and heavyweight athletes, suggesting that long-term differences in energy intake or metabolic demands may shape microbial structure. *Lactobacillus*, *Alistipes*, and *Fusobacterium* were more abundant in heavyweight athletes, whereas *Prevotella*, *Parabacteroides*, and *Veillonella* were enriched in lightweight athletes. These patterns likely reflect differences in dietary intake, with heavier athletes consuming more fermentable substrates that support carbohydrate-fermenting taxa. In contrast, taxa such as *Prevotella* and *Veillonella*, often linked to fiber metabolism and endurance [7,40,41], may indicate adaptations aligned with leaner body composition. We categorized athletes into lightweight (≤64 kg) and heavyweight (≥67 kg) groups to reflect practical distinctions in weight-class sports rather than arbitrary subdivisions. This grouping allowed us to explore potential microbiota differences associated with long-term energy intake and metabolic demands, thereby providing contextually relevant insights despite the modest cohort size. These microbial differences likely mirror long-term dietary and training adaptations in different weight classes. Beyond long-term weight-class differences, acute weight regulation also provided meaningful contrasts. Similarly, the pre- and post-competition weight-change groups (gain vs. loss) captured the actual direction of acute weight manipulation, a physiologically meaningful process in weight-class sports. This grouping enabled us to capture short-term microbial shifts associated with rapid body mass adjustments, thereby complementing the long-term perspective provided by weight-class comparisons and highlighting the dynamic nature of microbial responses to both chronic and acute weight regulation in athletes. These short-term microbial shifts likely reflect acute dietary and physiological adaptations during weight regulation in weight-class athletes.

Body weight changes before and after competition were associated with shifts in gut microbiota despite no significant differences in alpha diversity. Beta diversity analyses revealed distinct clustering between athletes who lost versus gained weight. Pre-competition weight gain (In_Pos) was linked to reduced abundance of SCFA-producing genera such as *Eubacterium*, *Alistipes*, and *Mitsuokella*, whereas post-competition weight gain (Off_Pos) showed higher relative abundance of *Fusobacterium*, *Klebsiella*, *Enterococcus*, and *Veillonella*. These findings indicate that rapid body mass manipulation can alter the relative abundance of specific taxa, even in the absence of changes in overall diversity.

Beyond these short-term effects, analysis of dietary energy intake clusters showed that Shannon diversity was significantly lower in the high-calorie group (G1) compared with the low-calorie group (G3), while species richness remained comparable. This reduction in diversity coincided with a higher relative abundance of *Lactobacillus* in G1 (adjusted *p* < 0.05), a genus widely recognized for its carbohydrate-fermenting capacity and role in gut health [19]. Together, these results highlight that both acute weight regulation and absolute caloric load influence gut microbial composition in weight-class athletes.

Collectively, these findings indicate that the gut microbiota of competitive weightlifters shows overall resilience at the community level, as phylum- and genus-level profiles remained stable across training phases. However, phase- and group-specific taxa still varied with protein intake, caloric load, weight regulation, and athlete size, pointing to candidate microbial markers of nutritional adaptation and metabolic stress. Integrating microbiome profiling with performance nutrition may inform strategies to support metabolic recovery, immune health, and overall well-being in weight-class athletes. These results highlight the complex interplay among training-phase dietary practices, body-weight regulation, and gut microbiota in competitive weightlifters. Although constrained by a modest sample size and single-season scope, the findings underscore practical implications for tailoring phase-specific dietary strategies and exploring microbiota-based monitoring to support recovery and performance. Taken together, they provide a foundation for applied research that connects microbiota changes with dietary planning and performance optimization across the competitive season, and they emphasize the need for larger, longitudinal studies to confirm these associations and extend their relevance across diverse athletic populations.

## 5. Conclusions

**Figure 7:**
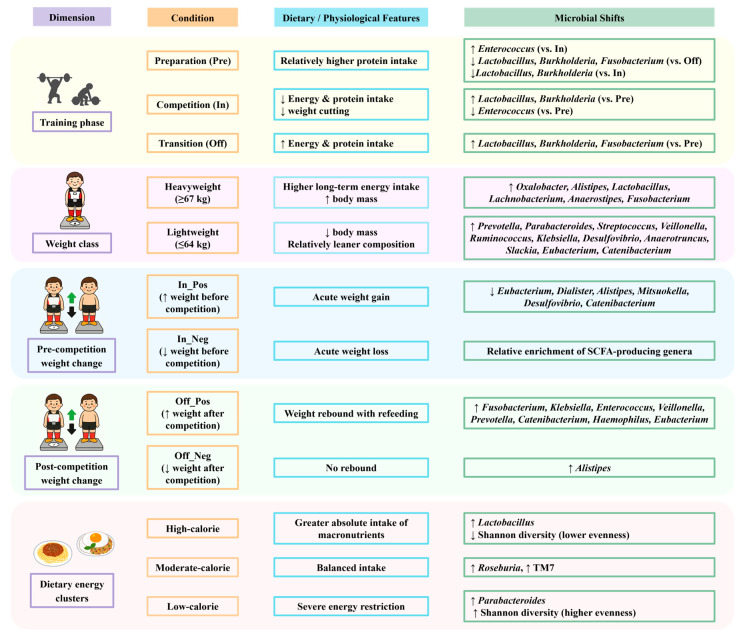
Summary of gut microbiota shifts in relation to training phases, weight classes, weight changes, and dietary intake clusters in competitive weightlifters. Arrows indicate relative increases (↑) or decreases (↓) in bacterial genera.

Although the overall gut microbiota composition of competitive weightlifters remained relatively stable across training phases, specific genera showed phase-related variation potentially reflecting differences in protein intake, energy availability, and post-competition recovery. *Enterococcus* was more abundant during the preparation phase, whereas *Lactobacillus* increased during the competition and transition phases, suggesting links to protein intake, energy availability, and post-competition recovery. Microbial profiles also varied by weight class, weight-change direction, and dietary energy clusters, with *Lactobacillus* enriched in the high-calorie group and *Megamonas* positively associated with carbohydrate intake. These multi-dimensional findings are summarized in Figure 7 to provide an integrated overview of microbiota shifts across conditions. Collectively, these findings highlight the interplay between dietary strategies, weight regulation, and the gut microbiota in weight-class athletes, underscoring the potential of microbiome monitoring to inform sports nutrition planning and athlete health management. These findings should be interpreted in light of the modest sample size and single-season scope, which limit generalizability. Future multi-center and longitudinal studies with larger cohorts will be essential to validate these associations and extend their application to performance optimization in diverse athletic populations.

## 6. Limitations

This study has several limitations. The sample size was modest, and one athlete’s missing competition data required exclusion of the corresponding preparation and transition records, resulting in unbalanced repeated measures (n = 10 in preparation, n = 12 in competition and transition). All data were collected within a single season at a single center, which limits generalizability. Microbiome profiling relied on 16S rRNA sequencing without functional or metabolic analyses, and no direct performance outcomes were assessed, restricting mechanistic inference. Body composition was measured with BIA rather than DXA, although standardized procedures were applied to ensure within-subject consistency. In terms of statistical analyses, multiple testing correction was applied only to high-dimensional taxa-level analyses (DESeq2) using the Benjamini–Hochberg procedure, while raw *p*-values were reported for alpha and beta diversity metrics. No covariates (e.g., sex, training load, or energy intake) were included in the PERMANOVA or ANOVA models. This decision was made because sex distribution did not differ significantly across phases, training load was not systematically recorded, and energy intake was analyzed as a primary variable of interest rather than a confounder. Additionally, PERMANOVA results were reported without corresponding effect size metrics (pseudo-F, R^2^), which limits the ability to quantify the proportion of variance explained by grouping factors. Moreover, although the study design was longitudinal, the incomplete data across phases precluded the use of a full repeated-measures statistical model. Therefore, one-way ANOVA was applied as a pragmatic approach to compare phase-specific differences. For these reasons, the findings should therefore be regarded as exploratory, yet the longitudinal design with synchronized dietary and fecal sampling and conservative statistics supports their internal validity. Larger multi-center studies with integrated functional and performance measures are warranted to confirm and extend these results.

## Figures and Tables

**Table 1:** Characteristic of study participants.

	Preparation (pre)	Competition (in)	Transition (off)	*p* Value
N (male/female)	10 (5/5)	12 (8/4)	12 (7/5)	0.731
Age (years)	21.0 ± 1.6	20.7 ± 1.5	20.8 ± 1.5	0.881
Body height (cm)	161.3 ± 9.0	163.4 ± 9.7	163.0 ± 9.8	0.864
Body weight (kg)	70.8 ± 18.5	73.8 ± 18.0	74.6 ± 17.1	0.873
Skeletal muscle mass (kg)	29.7 ± 6.0	32.3 ± 6.0	32.3 ± 6.1	0.518
Skeletal muscle mass (%)	42.5 ± 3.7	44.5 ± 4.9	43.8 ± 4.7	0.590
Fat mass (kg)	18.5 ± 9.7	17.2 ± 10.1	17.9 ± 9.8	0.951
Fat mass (%)	25.0 ± 6.8	22.1 ± 8.4	23.0 ± 8.2	0.678

Data are expressed as the mean ± SD.

**Table 2:** Macronutrient and energy intake of weightlifters during preparation, competition, and transition phases.

	Preparation (pre) n = 10	Competition (in) n = 11	Transition (off) n = 11	η^2^	*p* Value	Post Hoc
Carbohydrate (g/day)	160.2 ± 85.5	124.9 ± 62.7	177.9 ± 45.3	0.120	0.156	
Carbohydrate (g/kg/day)	2.3 ± 1.3	1.7 ± 0.8	2.5 ± 0.7	0.142	0.108	
Carbohydrate (% kcal/day)	45.1 ± 9.2	46.7 ± 6.9	41.7 ± 5.7	0.089	0.261	
Fat (g/day)	55.8 ± 28.1	40.0 ± 21.8	71.3 ± 18.1	0.277	0.009 **	in < off
Fat (g/kg/day)	0.8 ± 0.4	0.5 ± 0.3	1.0 ± 0.3	0.305	0.005 **	in < off
Fat (% kcal/day)	33.6 ± 5.1	34.4 ± 5.8	38.5 ± 3.8	0.181	0.055	
Protein (g/day)	78.8 ± 50.8	46.0 ± 25.2	80.4 ± 16.8	0.214	0.031 *	in < off
Protein (g/kg/day)	1.1 ± 0.5	0.6 ± 0.3	1.1 ± 0.3	0.299	0.006 **	in < pre, off
Protein (% kcal/day)	21.3 ± 6.6	17.5 ± 4.9	19.8 ± 2.2	0.107	0.195	
Calories (kcal/day)	1457.5 ± 693.8	1043.7 ± 526.9	1674.6 ± 387.0	0.220	0.027 *	in < off
Calories (kcal/kg/day)	20.9 ± 9.2	13.8 ± 6.6	23.4 ± 6.8	0.255	0.014 *	in < off

Data are expressed as the mean ± SD. * *p* < 0.05, ** *p* < 0.01, *** *p* < 0.001. effect size (η^2^).

**Table 3:** Blood biochemical parameters of weightlifters during preparation, competition, and transition phases.

	Preparation (pre) n = 10	Competition (in) n = 12	Transition (off) n = 12	η^2^	*p* Value	Post Hoc
GLU (mg/dL)	92.1 ± 6.8	82.4 ± 10.1	84.1 ± 6.0	0.229	0.018 *	in < pre
BUN (mg/dL)	16.2 ± 1.2	18.1 ± 2.2	18.0 ± 2.3	0.162	0.064	
Creatinine (mg/dL)	1.30 ± 0.03	1.29 ± 0.15	1.22 ± 0.24	0.039	0.541	
UA (mg/dL)	5.77 ± 0.33	6.14 ± 0.62	5.96 ± 0.71	0.065	0.353	
GOT (U/L)	27.5 ± 3.7	22.8 ± 8.9	24.2 ± 4.9	0.091	0.226	
GPT (U/L)	19.6 ± 2.5	18.5 ± 13.5	21.5 ± 10.8	0.016	0.776	
Lactate (mmol/L)	2.31 ± 0.36	1.39 ± 0.47	0.94 ± 0.11	0.736	<0.001 ***	off < in < pre
CPK (U/L)	445.1 ± 14.5	320.8 ± 178.4	369.0 ± 300.3	0.059	0.387	
LDH (U/L)	395.0 ± 38.1	341.5 ± 48.9	267.3 ± 47.8	0.585	<0.001 ***	off < in < pre
NH3 (μmol/L)	88.0 ± 5.0	53.5 ± 13.8	74.8 ± 22.8	0.456	<0.001 ***	in < pre, off

Data are expressed as the mean ± SD. * *p* < 0.05, ** *p* < 0.01, *** *p* < 0.001. effect size (η^2^), Glucose (GLU), blood urea nitrogen (BUN), creatinine, uric acid (UA), glutamic oxaloacetic transaminase (GOT), glutamic pyruvic transaminase (GPT), creatine phosphokinase (CPK), lactate dehydrogenase (LDH), and ammonia (NH_3_).

## Data Availability

The data presented in this study are available within the article.

## Supplementary Materials

The following supporting information can be downloaded at: https://www.mdpi.com/article/10.3390/nu17203199/s1, Table S1: DESeq2 analysis identifying differentially abundant genera between the samples obtained through pre and in; Table S2: DESeq2 analysis identifying differentially abundant genera between the samples obtained through in and off; Table S3: DESeq2 analysis identifying differentially abundant genera between the samples obtained through pre and off; Table S4: DESeq2 analysis identifying differentially abundant genera between the samples obtained through heavy and light; Table S5: DESeq2 analysis identifying differentially abundant genera between the samples obtained through In_Pos and In_Neg; Table S6: DESeq2 analysis identifying differentially abundant genera between the samples obtained through Off_Pos and Off_Neg; Table S7: DESeq2 analysis identifying differentially abundant genera between the samples obtained through G1 and G3.
